# [(*Z*)-*O*-Isopropyl *N*-(*m*-tol­yl)thio­carbamato-κ*S*](tricyclo­hexyl­phosphine-κ*P*)gold(I)

**DOI:** 10.1107/S160053681000975X

**Published:** 2010-03-20

**Authors:** Primjira P. Tadbuppa, Edward R. T. Tiekink

**Affiliations:** a Department of Chemistry, National University of Singapore, Singapore 117543; b Department of Chemistry, University of Malaya, 50603 Kuala Lumpur, Malaysia

## Abstract

The Au atom in the title compound, [Au(C_11_H_14_NOS)(C_18_H_33_P)], is coordinated within an *S*,*P*-donor set that defines a slightly distorted linear geometry [S—Au—P = 174.73 (3)°], with the distortion due in part to a close intra­molecular Au⋯O contact [3.060 (3) Å]. In the crystal structure, mol­ecules are arranged in layers in the *bc* plane with the primary connections between the arrays being of the type C—H⋯π.

## Related literature

For the structural systematics and luminescence properties of phosphinegold(I) carbonimidothio­ates, see: Ho *et al.* (2006 ▶); Ho & Tiekink (2007 ▶); Kuan *et al.* (2008 ▶). For the synthesis, see Hall *et al.* (1993 ▶).
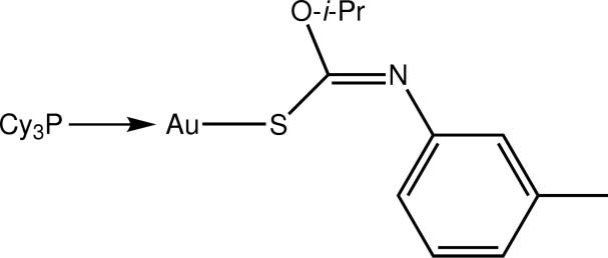


## Experimental

### 

#### Crystal data

[Au(C_11_H_14_NOS)(C_18_H_33_P)]
*M* *_r_* = 685.67
Monoclinic, 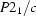

*a* = 13.7415 (18) Å
*b* = 13.3528 (18) Å
*c* = 16.788 (2) Å
β = 100.308 (3)°
*V* = 3030.7 (7) Å^3^
*Z* = 4
Mo *K*α radiation
μ = 5.00 mm^−1^
*T* = 223 K
0.37 × 0.10 × 0.10 mm

#### Data collection

Bruker SMART CCD diffractometer
Absorption correction: multi-scan (*SADABS*; Bruker, 2000 ▶) *T*_min_ = 0.445, *T*_max_ = 1.000
20885 measured reflections
6943 independent reflections
5710 reflections with *I* > 2σ(*I*)
*R*_int_ = 0.031

#### Refinement

*R*[*F* ^2^ > 2σ(*F* ^2^)] = 0.028
*wR*(*F* ^2^) = 0.072
*S* = 1.03
6943 reflections
308 parameters
H-atom parameters constrained
Δρ_max_ = 1.60 e Å^−3^
Δρ_min_ = −0.40 e Å^−3^

### 

Data collection: *SMART* (Bruker, 2000 ▶); cell refinement: *SAINT* (Bruker, 2000 ▶); data reduction: *SAINT*; program(s) used to solve structure: *PATTY* in *DIRDIF92* (Beurskens *et al.*, 1992 ▶); program(s) used to refine structure: *SHELXL97* (Sheldrick, 2008 ▶); molecular graphics: *ORTEP-3* (Farrugia, 1997 ▶) and *DIAMOND* (Brandenburg, 2006 ▶); software used to prepare material for publication: *publCIF* (Westrip, 2010 ▶).

## Supplementary Material

Crystal structure: contains datablocks global. DOI: 10.1107/S160053681000975X/hg2660sup1.cif

Structure factors: contains datablocks I. DOI: 10.1107/S160053681000975X/hg2660Isup2.hkl

Additional supplementary materials:  crystallographic information; 3D view; checkCIF report

## Figures and Tables

**Table 1:** Selected bond lengths (Å)

Au—P1	2.2692 (9)
Au—S1	2.3051 (9)

**Table 2:** Hydrogen-bond geometry (Å, °) *Cg* is the centroid of the C2–C7 ring.

*D*—H⋯*A*	*D*—H	H⋯*A*	*D*⋯*A*	*D*—H⋯*A*
C16—H16a⋯*Cg*^i^	0.98	2.98	3.655 (5)	127

Symmetry code: (i) 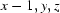.

**Fig. 1.**
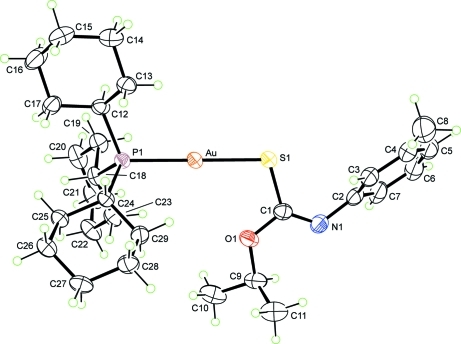
Molecular structure of (I) showing atom-labelling scheme and displacement ellipsoids at the 35% probability level.

**Fig. 2.**
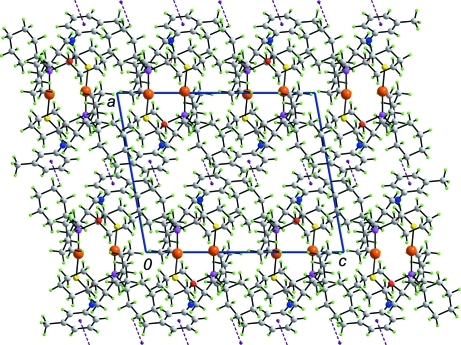
A view of the unit cell contents of (I) shown in projection down the b axis with the C–H···π interactions shown as purple dashed lines. Colour code: Au, orange; S, yellow; P, pink; O, red; N, blue; C, grey; and H, green.

## supplementary crystallographic information

### Comment

In the context of crystal engineering and luminescence studies of R_3_PAu[SC(OR')═NR''], for R, R' and R'' = alkyl and aryl, derivatives (Ho *et al.* 2006; Ho & Tiekink, 2007; Kuan *et al.*, 2008), the synthesis and characterisation of the title compound, (I), were investigated.

The gold atom in (I) exists within an *SP* donor set defined by the phosphine-*P* and thiolate-*S* atoms, Table 1 and Fig. 1. The carbonimidothioate ligand is coordinating as a thiolate ligand as evidenced by the magnitudes of the C1—S1 [1.766 (4) Å] and C1═N1 [1.266 (5) Å] bond distances. The coordination geometry is distorted from the ideal linear [S—Au—P = 174.73 (3) °] owing to the close approach of the O1 atom [3.060 (3) Å]. In the crystal structure, molecules forms layers in the *ab* plane with connections between the layers being of the type C–H···π, Table 2 and Fig. 2.

### Experimental

Compound (I) was prepared following the standard literature procedure from the reaction of Cy_3_PAuCl and (*i*-Pr)OC(═*S*)*N*(*H*)(*m*-tolyl) in the presence of NaOH (Hall *et al.*, 1993). Crystals were obtained by the slow evaporation of a CH_2_Cl_2_/hexane (3/1) solution held at room temperature.

### Refinement

The H atoms were geometrically placed (C—H = 0.94-0.99 Å) and refined as riding with *U*_iso_(H) = 1.2-1.5*U*_eq_(C). The maximum and minimum residual electron density peaks of 1.60 and 0.40 e Å^-3^, respectively, were located 0.84 Å and 1.53 Å from the Au atom.

### Crystal data

**Table:**

[Au(C_11_H_14_NOS)(C_18_H_33_P)]	*F*(000) = 1384
*M**_r_* = 685.67	*D*_x_ = 1.503 Mg m^−^^3^
Monoclinic, *P*2_1_/*c*	Mo *K*α radiation, λ = 0.71069 Å
Hall symbol: -P 2ybc	Cell parameters from 993 reflections
*a* = 13.7415 (18) Å	θ = 2.4–26.7°
*b* = 13.3528 (18) Å	µ = 5.00 mm^−^^1^
*c* = 16.788 (2) Å	*T* = 223 K
β = 100.308 (3)°	Block, colourless
*V* = 3030.7 (7) Å^3^	0.37 × 0.10 × 0.10 mm
*Z* = 4	

### Data collection

**Table:**

Bruker SMART CCD diffractometer	6943 independent reflections
Radiation source: fine-focus sealed tube	5710 reflections with *I* > 2σ(*I*)
graphite	*R*_int_ = 0.031
ω scans	θ_max_ = 27.5°, θ_min_ = 1.5°
Absorption correction: multi-scan (*SADABS*; Bruker, 2000)	*h* = −17→17
*T*_min_ = 0.445, *T*_max_ = 1.000	*k* = −12→17
20885 measured reflections	*l* = −21→20

### Refinement

**Table:**

Refinement on *F*^2^	Primary atom site location: structure-invariant direct methods
Least-squares matrix: full	Secondary atom site location: difference Fourier map
*R*[*F*^2^ > 2σ(*F*^2^)] = 0.028	Hydrogen site location: inferred from neighbouring sites
*wR*(*F*^2^) = 0.072	H-atom parameters constrained
*S* = 1.03	*w* = 1/[σ^2^(*F*_o_^2^) + (0.0386*P*)^2^] where *P* = (*F*_o_^2^ + 2*F*_c_^2^)/3
6943 reflections	(Δ/σ)_max_ = 0.001
308 parameters	Δρ_max_ = 1.60 e Å^−^^3^
0 restraints	Δρ_min_ = −0.40 e Å^−^^3^

### Special details

**Table:**

Geometry. All s.u.'s (except the s.u. in the dihedral angle between two l.s. planes) are estimated using the full covariance matrix. The cell s.u.'s are taken into account individually in the estimation of s.u.'s in distances, angles and torsion angles; correlations between s.u.'s in cell parameters are only used when they are defined by crystal symmetry. An approximate (isotropic) treatment of cell s.u.'s is used for estimating s.u.'s involving l.s. planes.
Refinement. Refinement of *F*^2^ against ALL reflections. The weighted *R*-factor wR and goodness of fit *S* are based on *F*^2^, conventional *R*-factors *R* are based on *F*, with *F* set to zero for negative *F*^2^. The threshold expression of *F*^2^ > 2σ(*F*^2^) is used only for calculating *R*-factors(gt) etc. and is not relevant to the choice of reflections for refinement. *R*-factors based on *F*^2^ are statistically about twice as large as those based on *F*, and *R*- factors based on ALL data will be even larger.

### Fractional atomic coordinates and isotropic or equivalent isotropic displacement parameters (Å^2^)

**Table:**

	*x*	*y*	*z*	*U*_iso_*/*U*_eq_	
Au	0.009245 (9)	0.077062 (9)	0.342984 (7)	0.03840 (5)	
S1	0.16091 (6)	0.15458 (6)	0.37674 (6)	0.0453 (2)	
P1	−0.13794 (6)	−0.00461 (6)	0.32160 (5)	0.03605 (18)	
O1	0.19821 (19)	0.00020 (18)	0.29116 (16)	0.0534 (6)	
N1	0.3294 (2)	0.1077 (2)	0.3217 (2)	0.0532 (8)	
C1	0.2414 (3)	0.0848 (2)	0.3270 (2)	0.0460 (8)	
C2	0.3764 (2)	0.1942 (3)	0.3601 (3)	0.0531 (9)	
C3	0.3875 (3)	0.2088 (3)	0.4421 (3)	0.0581 (10)	
H3	0.3594	0.1628	0.4738	0.070*	
C4	0.4409 (3)	0.2924 (4)	0.4799 (3)	0.0660 (11)	
C5	0.4820 (3)	0.3578 (3)	0.4314 (4)	0.0733 (13)	
H5	0.5176	0.4137	0.4549	0.088*	
C6	0.4720 (3)	0.3428 (4)	0.3502 (3)	0.0733 (13)	
H6	0.5005	0.3883	0.3184	0.088*	
C7	0.4203 (3)	0.2612 (3)	0.3142 (3)	0.0640 (11)	
H7	0.4148	0.2509	0.2582	0.077*	
C8	0.4511 (4)	0.3060 (5)	0.5685 (3)	0.0981 (17)	
H8A	0.5165	0.3317	0.5901	0.147*	
H8B	0.4418	0.2422	0.5937	0.147*	
H8C	0.4016	0.3531	0.5798	0.147*	
C9	0.2533 (3)	−0.0593 (3)	0.2416 (3)	0.0610 (11)	
H9	0.2894	−0.0142	0.2102	0.073*	
C10	0.1759 (4)	−0.1171 (4)	0.1845 (3)	0.0847 (15)	
H10A	0.1317	−0.0706	0.1515	0.127*	
H10B	0.1384	−0.1587	0.2155	0.127*	
H10C	0.2080	−0.1592	0.1498	0.127*	
C11	0.3256 (4)	−0.1286 (4)	0.2928 (3)	0.0811 (14)	
H11A	0.3755	−0.0893	0.3275	0.122*	
H11B	0.3572	−0.1708	0.2579	0.122*	
H11C	0.2907	−0.1701	0.3258	0.122*	
C12	−0.2309 (3)	0.0592 (2)	0.3696 (2)	0.0421 (8)	
H12	−0.2484	0.1216	0.3384	0.050*	
C13	−0.1880 (3)	0.0913 (3)	0.4560 (2)	0.0511 (9)	
H13A	−0.1282	0.1312	0.4558	0.061*	
H13B	−0.1692	0.0316	0.4892	0.061*	
C14	−0.2617 (3)	0.1526 (3)	0.4934 (3)	0.0649 (11)	
H14A	−0.2330	0.1686	0.5497	0.078*	
H14B	−0.2752	0.2158	0.4638	0.078*	
C15	−0.3573 (3)	0.0959 (4)	0.4909 (3)	0.0715 (13)	
H15A	−0.4048	0.1384	0.5121	0.086*	
H15B	−0.3449	0.0365	0.5256	0.086*	
C16	−0.4009 (3)	0.0637 (4)	0.4053 (3)	0.0778 (14)	
H16A	−0.4200	0.1232	0.3719	0.093*	
H16B	−0.4606	0.0238	0.4060	0.093*	
C17	−0.3273 (2)	0.0023 (3)	0.3681 (3)	0.0568 (10)	
H17A	−0.3135	−0.0606	0.3982	0.068*	
H17B	−0.3563	−0.0144	0.3120	0.068*	
C18	−0.1932 (2)	−0.0195 (2)	0.21456 (19)	0.0404 (7)	
H18	−0.2501	−0.0659	0.2110	0.048*	
C19	−0.2310 (3)	0.0793 (3)	0.1754 (2)	0.0519 (9)	
H19A	−0.1764	0.1275	0.1809	0.062*	
H19B	−0.2817	0.1067	0.2036	0.062*	
C20	−0.2749 (4)	0.0657 (3)	0.0859 (3)	0.0641 (12)	
H20A	−0.3348	0.0247	0.0810	0.077*	
H20B	−0.2940	0.1314	0.0620	0.077*	
C21	−0.2044 (3)	0.0170 (3)	0.0393 (2)	0.0670 (12)	
H21A	−0.1498	0.0631	0.0360	0.080*	
H21B	−0.2387	0.0032	−0.0160	0.080*	
C22	−0.1630 (4)	−0.0807 (3)	0.0790 (2)	0.0602 (11)	
H22A	−0.1121	−0.1066	0.0503	0.072*	
H22B	−0.2161	−0.1305	0.0742	0.072*	
C23	−0.1184 (3)	−0.0660 (3)	0.1681 (2)	0.0485 (9)	
H23A	−0.0966	−0.1308	0.1922	0.058*	
H23B	−0.0603	−0.0223	0.1726	0.058*	
C24	−0.1207 (2)	−0.1309 (2)	0.36852 (19)	0.0373 (7)	
H24	−0.1315	−0.1229	0.4249	0.045*	
C25	−0.1948 (2)	−0.2105 (2)	0.3298 (2)	0.0412 (7)	
H25A	−0.1854	−0.2234	0.2742	0.049*	
H25B	−0.2624	−0.1860	0.3278	0.049*	
C26	−0.1799 (3)	−0.3075 (3)	0.3788 (2)	0.0490 (8)	
H26A	−0.1947	−0.2955	0.4330	0.059*	
H26B	−0.2260	−0.3585	0.3524	0.059*	
C27	−0.0745 (3)	−0.3458 (3)	0.3862 (2)	0.0537 (9)	
H27A	−0.0660	−0.4060	0.4201	0.064*	
H27B	−0.0616	−0.3640	0.3324	0.064*	
C28	−0.0008 (3)	−0.2657 (3)	0.4234 (2)	0.0558 (10)	
H28A	0.0668	−0.2903	0.4253	0.067*	
H28B	−0.0096	−0.2522	0.4791	0.067*	
C29	−0.0154 (2)	−0.1691 (3)	0.3743 (2)	0.0477 (8)	
H29A	0.0312	−0.1183	0.4002	0.057*	
H29B	−0.0018	−0.1814	0.3198	0.057*	

### Atomic displacement parameters (Å^2^)

**Table:**

	*U*^11^	*U*^22^	*U*^33^	*U*^12^	*U*^13^	*U*^23^
Au	0.04093 (8)	0.03425 (8)	0.03913 (8)	−0.00328 (5)	0.00472 (5)	−0.00191 (5)
S1	0.0431 (4)	0.0385 (5)	0.0546 (5)	−0.0042 (4)	0.0096 (4)	−0.0074 (4)
P1	0.0378 (4)	0.0335 (4)	0.0355 (4)	0.0002 (3)	0.0028 (3)	−0.0008 (3)
O1	0.0592 (15)	0.0424 (14)	0.0606 (16)	−0.0018 (12)	0.0163 (12)	−0.0119 (12)
N1	0.0439 (17)	0.0524 (18)	0.064 (2)	0.0025 (15)	0.0108 (15)	0.0019 (15)
C1	0.050 (2)	0.039 (2)	0.047 (2)	0.0068 (15)	0.0062 (16)	0.0050 (14)
C2	0.0348 (17)	0.051 (2)	0.072 (3)	0.0081 (16)	0.0043 (17)	0.0029 (19)
C3	0.043 (2)	0.054 (2)	0.076 (3)	0.0027 (17)	0.0061 (19)	0.000 (2)
C4	0.043 (2)	0.071 (3)	0.079 (3)	0.006 (2)	0.001 (2)	−0.007 (2)
C5	0.048 (2)	0.054 (3)	0.113 (4)	0.0004 (19)	0.002 (3)	0.002 (3)
C6	0.050 (2)	0.065 (3)	0.101 (4)	−0.002 (2)	0.004 (2)	0.020 (3)
C7	0.040 (2)	0.066 (3)	0.084 (3)	0.0013 (19)	0.0061 (19)	0.016 (2)
C8	0.080 (3)	0.115 (5)	0.098 (4)	−0.010 (3)	0.013 (3)	−0.025 (3)
C9	0.075 (3)	0.046 (2)	0.066 (3)	0.010 (2)	0.025 (2)	−0.0031 (18)
C10	0.114 (4)	0.063 (3)	0.079 (3)	0.008 (3)	0.022 (3)	−0.022 (3)
C11	0.100 (4)	0.068 (3)	0.079 (3)	0.022 (3)	0.026 (3)	0.001 (2)
C12	0.0438 (19)	0.0367 (19)	0.0438 (19)	0.0081 (14)	0.0025 (15)	−0.0008 (14)
C13	0.054 (2)	0.052 (2)	0.048 (2)	0.0014 (17)	0.0096 (17)	−0.0102 (16)
C14	0.077 (3)	0.060 (3)	0.062 (3)	0.011 (2)	0.023 (2)	−0.010 (2)
C15	0.064 (3)	0.079 (3)	0.077 (3)	0.023 (2)	0.026 (2)	−0.006 (2)
C16	0.046 (2)	0.101 (4)	0.089 (4)	0.017 (2)	0.017 (2)	−0.005 (3)
C17	0.0386 (19)	0.067 (3)	0.063 (2)	0.0069 (18)	0.0053 (17)	−0.008 (2)
C18	0.0428 (17)	0.0397 (19)	0.0358 (17)	−0.0096 (14)	−0.0004 (13)	0.0027 (13)
C19	0.060 (2)	0.045 (2)	0.047 (2)	−0.0031 (17)	0.0004 (17)	0.0100 (15)
C20	0.077 (3)	0.055 (3)	0.051 (2)	−0.015 (2)	−0.014 (2)	0.0186 (18)
C21	0.096 (3)	0.061 (3)	0.039 (2)	−0.030 (2)	−0.001 (2)	0.0029 (18)
C22	0.092 (3)	0.052 (2)	0.035 (2)	−0.016 (2)	0.008 (2)	−0.0033 (16)
C23	0.061 (2)	0.046 (2)	0.0375 (19)	−0.0088 (17)	0.0063 (16)	−0.0052 (14)
C24	0.0418 (17)	0.0330 (17)	0.0365 (17)	−0.0006 (14)	0.0049 (13)	0.0002 (13)
C25	0.0474 (18)	0.0379 (19)	0.0371 (17)	−0.0019 (15)	0.0046 (14)	0.0002 (13)
C26	0.063 (2)	0.0391 (19)	0.045 (2)	−0.0057 (17)	0.0084 (17)	0.0003 (15)
C27	0.077 (3)	0.0328 (19)	0.052 (2)	0.0086 (18)	0.0120 (19)	0.0050 (15)
C28	0.058 (2)	0.044 (2)	0.063 (2)	0.0100 (18)	0.0037 (19)	0.0075 (18)
C29	0.0426 (18)	0.041 (2)	0.058 (2)	0.0021 (15)	0.0032 (16)	0.0055 (16)

### Geometric parameters (Å, °)

**Table:**

Au—P1	2.2692 (9)	C15—C16	1.517 (7)
Au—S1	2.3051 (9)	C15—H15A	0.9800
S1—C1	1.766 (4)	C15—H15B	0.9800
P1—C12	1.837 (4)	C16—C17	1.521 (5)
P1—C18	1.832 (3)	C16—H16A	0.9800
P1—C24	1.858 (3)	C16—H16B	0.9800
O1—C1	1.365 (4)	C17—H17A	0.9800
O1—C9	1.456 (5)	C17—H17B	0.9800
N1—C1	1.266 (5)	C18—C19	1.524 (5)
N1—C2	1.420 (5)	C18—C23	1.529 (5)
C2—C3	1.372 (6)	C18—H18	0.9900
C2—C7	1.387 (6)	C19—C20	1.527 (5)
C3—C4	1.421 (6)	C19—H19A	0.9800
C3—H3	0.9400	C19—H19B	0.9800
C4—C5	1.383 (6)	C20—C21	1.498 (7)
C4—C8	1.480 (7)	C20—H20A	0.9800
C5—C6	1.359 (7)	C20—H20B	0.9800
C5—H5	0.9400	C21—C22	1.529 (6)
C6—C7	1.379 (6)	C21—H21A	0.9800
C6—H6	0.9400	C21—H21B	0.9800
C7—H7	0.9400	C22—C23	1.525 (5)
C8—H8A	0.9700	C22—H22A	0.9800
C8—H8B	0.9700	C22—H22B	0.9800
C8—H8C	0.9700	C23—H23A	0.9800
C9—C11	1.509 (6)	C23—H23B	0.9800
C9—C10	1.511 (7)	C24—C29	1.522 (4)
C9—H9	0.9900	C24—C25	1.534 (4)
C10—H10A	0.9700	C24—H24	0.9900
C10—H10B	0.9700	C25—C26	1.529 (5)
C10—H10C	0.9700	C25—H25A	0.9800
C11—H11A	0.9700	C25—H25B	0.9800
C11—H11B	0.9700	C26—C27	1.521 (5)
C11—H11C	0.9700	C26—H26A	0.9800
C12—C17	1.524 (5)	C26—H26B	0.9800
C12—C13	1.527 (5)	C27—C28	1.528 (5)
C12—H12	0.9900	C27—H27A	0.9800
C13—C14	1.522 (5)	C27—H27B	0.9800
C13—H13A	0.9800	C28—C29	1.525 (5)
C13—H13B	0.9800	C28—H28A	0.9800
C14—C15	1.511 (6)	C28—H28B	0.9800
C14—H14A	0.9800	C29—H29A	0.9800
C14—H14B	0.9800	C29—H29B	0.9800
			
P1—Au—S1	174.73 (3)	C15—C16—H16B	109.4
C1—S1—Au	105.20 (13)	C17—C16—H16B	109.4
C12—P1—C18	106.37 (16)	H16A—C16—H16B	108.0
C12—P1—C24	106.32 (16)	C12—C17—C16	111.4 (3)
C18—P1—C24	108.42 (15)	C12—C17—H17A	109.4
C12—P1—Au	112.19 (12)	C16—C17—H17A	109.4
C18—P1—Au	114.12 (11)	C12—C17—H17B	109.4
C24—P1—Au	109.07 (10)	C16—C17—H17B	109.4
C1—O1—C9	118.3 (3)	H17A—C17—H17B	108.0
C1—N1—C2	121.8 (3)	C19—C18—C23	109.9 (3)
N1—C1—O1	121.0 (3)	C19—C18—P1	112.2 (2)
N1—C1—S1	126.4 (3)	C23—C18—P1	110.2 (2)
O1—C1—S1	112.7 (3)	C19—C18—H18	108.1
C3—C2—N1	122.1 (4)	C23—C18—H18	108.1
C3—C2—C7	119.0 (4)	P1—C18—H18	108.1
N1—C2—C7	118.7 (4)	C18—C19—C20	111.4 (3)
C2—C3—C4	121.1 (4)	C18—C19—H19A	109.3
C2—C3—H3	119.5	C20—C19—H19A	109.3
C4—C3—H3	119.5	C18—C19—H19B	109.3
C5—C4—C3	117.7 (5)	C20—C19—H19B	109.3
C5—C4—C8	122.7 (5)	H19A—C19—H19B	108.0
C3—C4—C8	119.6 (5)	C21—C20—C19	112.8 (4)
C6—C5—C4	121.3 (5)	C21—C20—H20A	109.0
C6—C5—H5	119.4	C19—C20—H20A	109.0
C4—C5—H5	119.4	C21—C20—H20B	109.0
C5—C6—C7	120.5 (5)	C19—C20—H20B	109.0
C5—C6—H6	119.7	H20A—C20—H20B	107.8
C7—C6—H6	119.7	C20—C21—C22	111.6 (3)
C6—C7—C2	120.5 (5)	C20—C21—H21A	109.3
C6—C7—H7	119.8	C22—C21—H21A	109.3
C2—C7—H7	119.8	C20—C21—H21B	109.3
C4—C8—H8A	109.5	C22—C21—H21B	109.3
C4—C8—H8B	109.5	H21A—C21—H21B	108.0
H8A—C8—H8B	109.5	C21—C22—C23	111.8 (3)
C4—C8—H8C	109.5	C21—C22—H22A	109.3
H8A—C8—H8C	109.5	C23—C22—H22A	109.3
H8B—C8—H8C	109.5	C21—C22—H22B	109.3
O1—C9—C11	111.5 (4)	C23—C22—H22B	109.3
O1—C9—C10	105.2 (4)	H22A—C22—H22B	107.9
C11—C9—C10	111.4 (4)	C18—C23—C22	111.4 (3)
O1—C9—H9	109.5	C18—C23—H23A	109.4
C11—C9—H9	109.5	C22—C23—H23A	109.4
C10—C9—H9	109.5	C18—C23—H23B	109.4
C9—C10—H10A	109.5	C22—C23—H23B	109.4
C9—C10—H10B	109.5	H23A—C23—H23B	108.0
H10A—C10—H10B	109.5	C29—C24—C25	110.2 (3)
C9—C10—H10C	109.5	C29—C24—P1	112.3 (2)
H10A—C10—H10C	109.5	C25—C24—P1	115.1 (2)
H10B—C10—H10C	109.5	C29—C24—H24	106.2
C9—C11—H11A	109.5	C25—C24—H24	106.2
C9—C11—H11B	109.5	P1—C24—H24	106.2
H11A—C11—H11B	109.5	C24—C25—C26	110.1 (3)
C9—C11—H11C	109.5	C24—C25—H25A	109.6
H11A—C11—H11C	109.5	C26—C25—H25A	109.6
H11B—C11—H11C	109.5	C24—C25—H25B	109.6
C17—C12—C13	110.0 (3)	C26—C25—H25B	109.6
C17—C12—P1	115.6 (2)	H25A—C25—H25B	108.1
C13—C12—P1	111.5 (2)	C27—C26—C25	111.3 (3)
C17—C12—H12	106.4	C27—C26—H26A	109.4
C13—C12—H12	106.4	C25—C26—H26A	109.4
P1—C12—H12	106.4	C27—C26—H26B	109.4
C12—C13—C14	111.8 (3)	C25—C26—H26B	109.4
C12—C13—H13A	109.3	H26A—C26—H26B	108.0
C14—C13—H13A	109.3	C28—C27—C26	110.5 (3)
C12—C13—H13B	109.3	C28—C27—H27A	109.6
C14—C13—H13B	109.3	C26—C27—H27A	109.6
H13A—C13—H13B	107.9	C28—C27—H27B	109.6
C15—C14—C13	111.0 (3)	C26—C27—H27B	109.6
C15—C14—H14A	109.4	H27A—C27—H27B	108.1
C13—C14—H14A	109.4	C27—C28—C29	110.9 (3)
C15—C14—H14B	109.4	C27—C28—H28A	109.5
C13—C14—H14B	109.4	C29—C28—H28A	109.5
H14A—C14—H14B	108.0	C27—C28—H28B	109.5
C14—C15—C16	111.3 (4)	C29—C28—H28B	109.5
C14—C15—H15A	109.4	H28A—C28—H28B	108.0
C16—C15—H15A	109.4	C24—C29—C28	110.5 (3)
C14—C15—H15B	109.4	C24—C29—H29A	109.6
C16—C15—H15B	109.4	C28—C29—H29A	109.6
H15A—C15—H15B	108.0	C24—C29—H29B	109.6
C15—C16—C17	111.3 (4)	C28—C29—H29B	109.6
C15—C16—H16A	109.4	H29A—C29—H29B	108.1
C17—C16—H16A	109.4		
			
P1—Au—S1—C1	107.5 (3)	C13—C14—C15—C16	−55.5 (5)
S1—Au—P1—C12	74.2 (3)	C14—C15—C16—C17	55.8 (5)
S1—Au—P1—C18	−164.8 (3)	C13—C12—C17—C16	55.6 (4)
S1—Au—P1—C24	−43.3 (3)	P1—C12—C17—C16	−177.1 (3)
C2—N1—C1—O1	−178.5 (3)	C15—C16—C17—C12	−56.1 (5)
C2—N1—C1—S1	2.8 (6)	C12—P1—C18—C19	53.4 (3)
C9—O1—C1—N1	−4.5 (5)	C24—P1—C18—C19	167.4 (3)
C9—O1—C1—S1	174.3 (3)	Au—P1—C18—C19	−70.8 (3)
Au—S1—C1—N1	171.7 (3)	C12—P1—C18—C23	176.2 (2)
Au—S1—C1—O1	−7.1 (3)	C24—P1—C18—C23	−69.8 (3)
C1—N1—C2—C3	58.2 (5)	Au—P1—C18—C23	52.0 (2)
C1—N1—C2—C7	−127.9 (4)	C23—C18—C19—C20	55.8 (4)
N1—C2—C3—C4	175.5 (3)	P1—C18—C19—C20	178.8 (3)
C7—C2—C3—C4	1.6 (5)	C18—C19—C20—C21	−54.8 (5)
C2—C3—C4—C5	−0.6 (6)	C19—C20—C21—C22	52.5 (4)
C2—C3—C4—C8	179.7 (4)	C20—C21—C22—C23	−52.9 (5)
C3—C4—C5—C6	−0.1 (6)	C19—C18—C23—C22	−56.7 (4)
C8—C4—C5—C6	179.5 (4)	P1—C18—C23—C22	179.2 (2)
C4—C5—C6—C7	−0.1 (7)	C21—C22—C23—C18	55.4 (5)
C5—C6—C7—C2	1.1 (6)	C12—P1—C24—C29	−147.5 (2)
C3—C2—C7—C6	−1.8 (6)	C18—P1—C24—C29	98.5 (3)
N1—C2—C7—C6	−175.9 (3)	Au—P1—C24—C29	−26.4 (3)
C1—O1—C9—C11	82.5 (5)	C12—P1—C24—C25	85.2 (3)
C1—O1—C9—C10	−156.6 (4)	C18—P1—C24—C25	−28.8 (3)
C18—P1—C12—C17	62.6 (3)	Au—P1—C24—C25	−153.6 (2)
C24—P1—C12—C17	−52.8 (3)	C29—C24—C25—C26	57.6 (4)
Au—P1—C12—C17	−172.0 (2)	P1—C24—C25—C26	−174.1 (2)
C18—P1—C12—C13	−170.8 (2)	C24—C25—C26—C27	−57.0 (4)
C24—P1—C12—C13	73.8 (3)	C25—C26—C27—C28	56.3 (4)
Au—P1—C12—C13	−45.4 (3)	C26—C27—C28—C29	−56.4 (4)
C17—C12—C13—C14	−55.7 (4)	C25—C24—C29—C28	−58.0 (4)
P1—C12—C13—C14	174.7 (3)	P1—C24—C29—C28	172.1 (3)
C12—C13—C14—C15	55.9 (5)	C27—C28—C29—C24	57.5 (4)

### Hydrogen-bond geometry (Å, °)

**Table:**

*D*—H···*A*	*D*—H	H···*A*	*D*···*A*	*D*—H···*A*
C16—H16a···Cg^i^	0.98	2.98	3.655 (5)	127

Symmetry codes: (i) *x*−1, *y*, *z*.
